# Role of dihydrotestosterone (DHT) on TGF-β1 signaling pathway in epithelial ovarian cancer cells

**DOI:** 10.1007/s00432-015-1998-y

**Published:** 2015-06-20

**Authors:** Karla Kohan-Ivani, Fernando Gabler, Alberto Selman, Margarita Vega, Carmen Romero

**Affiliations:** Laboratory of Endocrinology and Reproductive Biology, University of Chile Clinical Hospital, Avda. Santos Dumont # 999, Independencia, Santiago, Chile; Department of Pathology, School of Medicine, San Borja Arriarán Clinical Hospital, University of Chile, Santiago, Chile; Department of Obstetrics and Gynecology, School of Medicine, University of Chile Clinical Hospital, Santiago, Chile; Advanced Center for Chronic Diseases (ACCDiS), School of Medicine, University of Chile, Santiago, Chile

**Keywords:** Epithelial ovarian cancer, Androgen receptor, TGF-β signaling pathway

## Abstract

**Purpose:**

One of the hypotheses regarding the genesis of epithelial ovarian cancer involves the action of androgens on the proliferation of epithelial ovarian cells, as well as inclusion cysts. The purpose of the present study was to evaluate whether DHT causes changes in the TGF-β1 pathway that might modify the anti-proliferative effect of the latter.

**Methods:**

The levels of TGF-β1 protein, of its receptors (TGFBR1 and TGFBR2), of Smad2/3 (canonical signaling pathway protein) and of p21 (cell cycle protein) were assessed in ovarian tissues, epithelial ovarian cancer cell lines (A2780) and control cell lines (HOSE) through the use of immunohistochemistry and immunocytochemistry. Additionally, cell lines were treated with 100 nmol/L DHT, 10 ng/mL of TGF-β1 and DHT + TGF-β1 during 72 h in the presence and absence of a siRNA against androgen receptor. After treatment, TGFBR1 and TGFBR2 levels were detected through Western blotting and p21 was assessed through immunocytochemistry.

**Results:**

Epithelial ovarian cancer tissues showed a decrease in TGF-β1 I receptor (*p* < 0.05) and a change in Smad2/3 protein levels. Additionally, after treatment of cell lines with DHT, protein levels of TGF-β1 receptors (TGFBR1–TGFBR2) showed a decrease (*p* < 0.05) that might cause a potential disorder in TGF-β1 response, represented by the significant decrease in p21 protein levels in the presence of DHT (*p* < 0.001).

**Conclusions:**

Overall, our results indicate a defect in the canonical TGF-β signaling pathway in epithelial ovarian cancer caused by androgen action, thus suggesting eventual changes in such tissue proliferation rates.

## Introduction

Epithelial ovarian cancer (EOC) accounts for 85–95 % of ovarian malignant neoplasms (Auersperg 2013). Epithelial ovarian cancer represents the second cause of gynecological death following uterine cervix cancer. According to a recent FIGO report, EOC is the seventh most common disease among women worldwide (Prat 2007). Among the features of EOC are a silent evolution, a late detection, a poor response to therapy and a high angiogenic potential. Consequently, women with EOC have a low survival rate (Jordan et al. 2006; Auersperg et al. 2002). The pathogenesis of ovarian carcinoma is somewhat unclear, and several mechanisms have been suggested to explain the etiology of such disease (Lukanova and Kaaks 2005). In such context, among the hypotheses attempting to explain its origin, there is one related to steroid hormones, proposing that excess stimulation of ovarian surface epithelium with androgens might increase cancer risk (Risch 1998). With regard to androgens and EOC, studies explain that androgens and other sex steroids have a significant role in ovarian carcinogenesis (Schock et al. 2014; Ligr et al. 2011; Olsen et al. 2008). To elucidate the molecular mechanism underlying the involvement of androgens in ovarian carcinogenesis, studies have linked the androgen signaling pathway with molecules such as the transforming growth factor beta 1 (TGF-β1) (Li and Karlan 2008). TGF-β1 is a member of a cytokine family known as a potent cell growth inhibitor. It also participates in a variety of cell processes such as cell proliferation, morphogenesis, migration and apoptosis (Antony et al. 2010; Siegel and Massagué 2003). The canonical TGF-β1 signaling pathway starts when the ligand (TGF-β1) binds the TGF-β1 II receptor (TGFBR2) that in turn recruits TGF-β1 I receptor (TGFBR1) creating a complex. TGFBR2 phosphorylates TGFBR1 at the GS domain, thus enabling its activation. Once TGFBR1 becomes activated, such receptor phosphorylates R-Smad (Smad2 and 3). Phosphorylation of Smad2/3 enables the creation of dimers or trimers with a co-Smad, Smad4. Such complex is translocated to the nucleus and interacts with the DNA-binding domain, recruiting co-activators or co-repressors to modulate gene transcription (Siegel and Massagué 2003). The TGF-β1 signaling pathway (Smad2, 3 and 4) is involved in the regulation of tissue homeostasis and is responsible for transcriptional activation of p21Cip1, p15INK4B and c-myc genes. The first two encode the cell cycle inhibitors p21 and p15, while c-myc is a transcriptional repressor (Massague and Gomis 2006). Studies on prostate cancer have demonstrated androgenic control in TGF-β1 signaling through suppression of TGFBR2 transcription suppression (Song et al. 2008). Another study, on prostate epithelial cells, showed that androgens such as DHT may generate a downregulation of Smad3 (Song et al. 2010). On the other hand, a relationship between the androgen receptor (AR) and TGF-β1 receptor expression has been demonstrated in ovarian cancer (Evangelou et al. 2000); however, the underlying mechanism is incompletely understood. Moreover, how such association might affect the levels of some molecules involved in proliferation remains unclear. Based on the fact that androgens play an important role in the development of ovarian cancer (Gibson et al. 2014), the purpose of the present study was to assess the role of androgen receptors and the androgen DHT on the canonical TGF-β1 signaling pathway through the assessment of a possible effect on molecules involved in proliferation such as the p21 protein.

## Materials and methods

The present research was approved by the Ethics Committee of the Hospital Clínico de la Universidad de Chile and by the Ethics Committee of the Escuela de Medicina de la Universidad de Chile. All study subjects provided a written informed consent.

### Human tissue specimens

Human ovarian specimens were obtained from fourteen (14) patients as follows: normal ovary samples (inactive ovaries, I-Ov) were obtained from women with a non-ovarian pathological condition (*n* = 7), and another specimen group consisted from samples obtained by women with a diagnosis of serous EOC (*n* = 7).

An experienced pathologist performed histopathological analysis and classification of samples. All the subjects participating in the study were perimenopausal or postmenopausal women, aged between 46 and 68.

### Immunohistochemistry

Immunostains for AR, TGF-β1, TGFBR1, TGFBR2, pSmad2 and pSmad3 were performed on 5-mm sections of paraffin-embedded ovarian tissue biopsies. Tissue sections were deparaffinized in xylene and hydrated in serial grading of alcohol solutions. Sections were incubated in an antigen recovery solution (sodium citrate buffer 10 mMol/L, pH 6.0) at 96–98 °C for 20 min. The endogenous peroxidase activity was blocked through incubation of the samples in hydrogen peroxide 3 % (v/v) for 15 min. Non-specific antibody binding was prevented with a specific blocker of the Histostain SP kit (Zymed Laboratories, San Francisco, CA, USA). Samples were incubated overnight at 4 °C with the primary antibody (Table 1). Negative controls were performed on adjacent sections and were incubated without the primary antibody, as well as with specific antisera of the non-immune species. The secondary antibody was a biotinylated anti-mouse/anti-rabbit immunoglobulin. The reaction was developed through the use of streptavidin–peroxidase system using 3,3′-diaminobenzidine as chromogen. Counterstaining was done with hematoxylin. Slides were analyzed under an optical Olympus BX51 microscope (Olympus Corporation, Tokyo, Japan). Images were acquired with a Micro-editor 3.3 RTV camera (Q Imaging, Surrey, BC, Canada). Immunohistochemical assessment of each protein was performed with a semiquantitative method previously described for endometrial tissue (Rivero et al. 2012). With the Image-Pro Plus^®^ acquisition system, images were acquired in 1000× resolution and processed in TIFF format. Analysis was performed through the measurement of positive pixel intensity per area using the semiquantitative integrated optical density tool (IOD) in the Image-Pro Plus 6.2 program (Media Cybernetics Inc., Silver Spring, MD, USA). IOD assessment was done randomly in different regions of the epithelium in each specimen. Data are presented as IOD arbitrary units (AU). The means of the values obtained per sample and per study group were expressed as mean ± SEM.

**Table 1:** Antibody and dilutions used in immunohistochemistry and immunocytochemistry

Antibody	Dilution	Supplier	Type
AR	1:300	Abcam	Rabbit polyclonal
TGF-β1	1:300	Abcam	Rabbit polyclonal
TGFBR1	1:200	Abcam	Rabbit polyclonal
TGFBR2	1:250	Abcam	Rabbit polyclonal
pSmad2	1:200	Abcam	Rabbit polyclonal
pSmad3	1:200	Abcam	Rabbit polyclonal

### Cell lines and cell culture

Normal human ovarian superficial epithelial cells (HOSE) obtained from a postmenopausal patient with endometrial cancer were immortalized with SV40-Tag. Conversely, A2780 is a drug-sensitive human ovarian cancer cell line with epithelial morphology that was established from tumor tissue of a patient before treatment. Both cell lines were cultured in DMEM-Ham/F12 medium (Sigma-Aldrich Co. St Louis, MO, USA) in the presence of penicillin G 100 IU/mL, streptomycin sulfate 100 mg/mL and amphotericin B with 10 % bovine serum treated with charcoal/dextrane (Hyclone™ Thermo Fisher Scientific, Rochester, NY, USA) until culture reached 80 % confluence, as previously described (Tapia et al. 2011).

### Cell culture treatment

Cells were washed twice in Dulbecco’s phosphate-buffered saline (DPBS, GIBCO^®^ Invitrogen Corporation, Camarillo, CA, USA) and cultured in serum-free medium with 0, 10 and 100 nmol/L DHT (Sigma-Aldrich Co, St Louis, MO, USA) during 72 h and with 10 ng/mL TGF-β1 (Abcam, Cambridge, MA, EE.UU.). In some experiments, cells were treated with DHT plus TGF-β1.

### Immunocytochemistry

HOSE and A2780 cells were fixed with 4 % paraformaldehyde during 15 min at room temperature. Endogenous peroxidase activity was blocked with sample incubation in hydrogen peroxide 3 % (v/v) during 15 min. Cells were washed and subsequently incubated with skim milk for 10 min at room temperature to block non-specific binding and were incubated overnight at 4 °C with the primary antibody against AR, TGF-β1, TGFBR1, TGFBR2, pSmad2, pSmad3 and p21. Subsequently, cells were washed and incubated with the secondary antibody (KPL, Kirkegaard & Perry Laboratories Inc, MD, USA) during 30 min at 37 °C. Cells were incubated during 1 min at room temperature with the 3,3′-diaminobenzidine liquid substrate (DAB) (DakoCytomation, Inc., CA, USA), and hematoxylin was used for counterstaining (Lerner Laboratories, Pittsburgh, PA, USA). Slides were analyzed using an Olympus BX51 optical microscope (Olympus Corporation, Tokyo, Japan). A Micro-editor 3.3 RTV camera (Q Imaging, Surrey, BC, Canada) was used for image acquisition. Each slide was analyzed using the measurement of positive pixel intensity by means of the integrated optical density semiquantitative analysis tool (IOD) in the 6,2 Image-Pro Plus program (Media Cybernetics Inc., Silver Spring, MD, USA). Data on p21 were expressed as positive cell percentage. Three independent observers carried out the analysis, and positive stain was assessed in at least 1000 cells per sample.

### Protein extraction and Western blotting

Cell cultures (roughly 10^6^ cells) underwent homogenization in a lysis buffer (Tris 50 mM, NaCl 150 mM, DOT 0.5 %, Triton 1 %, SDS 0.1 %). After spinning at 15,000×*g* for 20 min, protein concentrations were quantified with the BCA protein assay kit (Pierce, Rockford, IL, USA). Subsequently, 40 g of protein was denatured and underwent fractionation in SDS-PAGE, to be finally transferred to a nitrocellulose membrane. After blocking with 10 % skim milk, membranes were incubated overnight with the primary antibody (TGFBR1 and TGFBR2) in TBST solution at 4 °C. After washing, membranes were incubated for 60 min at room temperature with a species-specific peroxidase-conjugated anti-rabbit IgG (KPL, Kirkegaard & Perry Laboratories Inc, MD, USA) at a 1:5000 dilution. After washing, bound antibodies were detected with an enhanced chemiluminescence system (Amersham Biosciences, Piscataway, NJ, USA). Then, membranes were stripped, washed and blocked. Subsequently, they were incubated with anti-GAPDH (Sigma-Aldrich Co, St Louis, MO, USA) at a 1:15,000 dilution during 1 h, washed and incubated with anti-mouse antibody (Abcam, Cambridge, MA, USA) at a 1:5,000 dilution for 30 min. At the end, bound antibodies were detected with chemiluminescence. Protein band intensities were quantified with the UN-SCAN-IT Automated Digitizing System Software version 5.1. Results were expressed as arbitrary units (AU). The means of values obtained per sample and per study group were expressed as mean ± SEM.

### Small-interference RNA constructs and transfection

Sequences used against human AR (GenBank adhesion no. NM_000044) were used in a prior study (Cai et al. 2011). Specifically, the sequence used for AR was 5′-ACCGAGGAGCUUUCCAGAAUCUGUU-3′. A non-specific control small-interference RNA (siRNA) (5′-CCAUGGCGCCAAUUCCAAACAGUUU-3′) was included in all the experiments. All transfections were carried out using siRNA Lipofectamine 2000 (Invitrogen) as per manufacturer’s instructions. Briefly, 500,000 A2780 cells were seeded in phenol red-free DMEM-Ham F12 medium containing 2 % bovine saline without antibiotics. Twenty-four (24) hours later, when cells were attached to the plate, they were transfected with siRNA (100 pmol) using 5 μL of Lipofectamine 2000 in a total volume of 1 mL of OPTI-MEM medium (Invitrogen) per well. Six (6) hours after transfection, transfection reagents were removed and cells were treated as indicated in each experiment. Western blotting was used to verify AR knockdown.

### RNA isolation and semiquantitative RT-PCR

Total RNA was isolated from A2780 cell line using TRIzol^®^ reagent as per manufacturer’s instructions. Quantitation of RNA was carried out with spectrophotometry (A260:A280), while RNA integrity was determined by denaturing agarose–formaldehyde gel electrophoresis. RNA was visualized by adding GelRed™ to the sample before loading on the gel. RNA was stored at −80 °C until used. Two micrograms (2 μg) of total RNA was digested with DNase I and was transcribed to cDNA through RT with M-MLV RT using random primers in a total volume of 25 mL. Amplifications were obtained through PCR using gene-specific primers (Table 2). GAPDH was used as an internal control. Semiquantitative RT-PCRs were achieved in the exponential linear zone amplification for each gene studied. PCR conditions for each gene were 2 mmol/L MgCl2, 0.20 mmol/L dNTPs, 2 U Taq DNA polymerase and 30 pmol of each primer. PCR amplification was carried out in a PTC-100 thermocycler (MJ Research Inc., Watertown, MA, USA) and a Mastercycler Personal (Eppendorf AG, Foster City, CA, USA). Resolution of PCR products was carried out on 2 % agarose gel electrophoresis with subsequent staining with GelRed™. Bands were analyzed with a UN-SCAN-IT gel 4.1 image analyzer (Silk Scientific Corporation) and normalized by GAPDH. Results were expressed in arbitrary units (AU). The means of the values obtained per sample and per study group were expressed as mean ± SEM.

**Table 2:** Gene sequence and PCR products

Gene	Sequence	bp
TGFBR1	5′ GGT CTT GCC CAT CTT CAC AT 3′ 5′ TCT GTG GCT GAA TCA TGT CT 3′	155
TGFBR2	5′ GTC TAC TCC ATG GCT CGT GT 3′ 5′ ATC TGG ATG CCC TGG TGG TT 3′	197
AR	5′ AGA TGG GCT TGA CTT CAG AAA G 3′ 5′ ATG GCT ATT CAG TAC TCC TGG A 3′	545
GAPDH	5′ GAG TCA ACG GAT TTG GTC GT 3′ 5′ ATC CAC AGT CTT CTG GGT G 3′	548
GAPDH	5′ CCA CCA TGG AGA AGG CTG GG 3′ 5′ ATC ACG CCA CAG TTT CCC GG 3′	287

### Statistical analysis

The number of ovarian tissue samples (*n* = 7 for each study group) was determined assuming 5 % probability of type 1 error and 20 % probability of type 2 error. Differences between groups were analyzed through the Kruskal–Wallis test and the Dunn’s post-test. All *p* values under 0.05 were considered significant.

## Results

Protein levels of AR and molecules of the TGF-β1 signaling pathway in ovarian epithelial tissue.

**Fig. 1:**
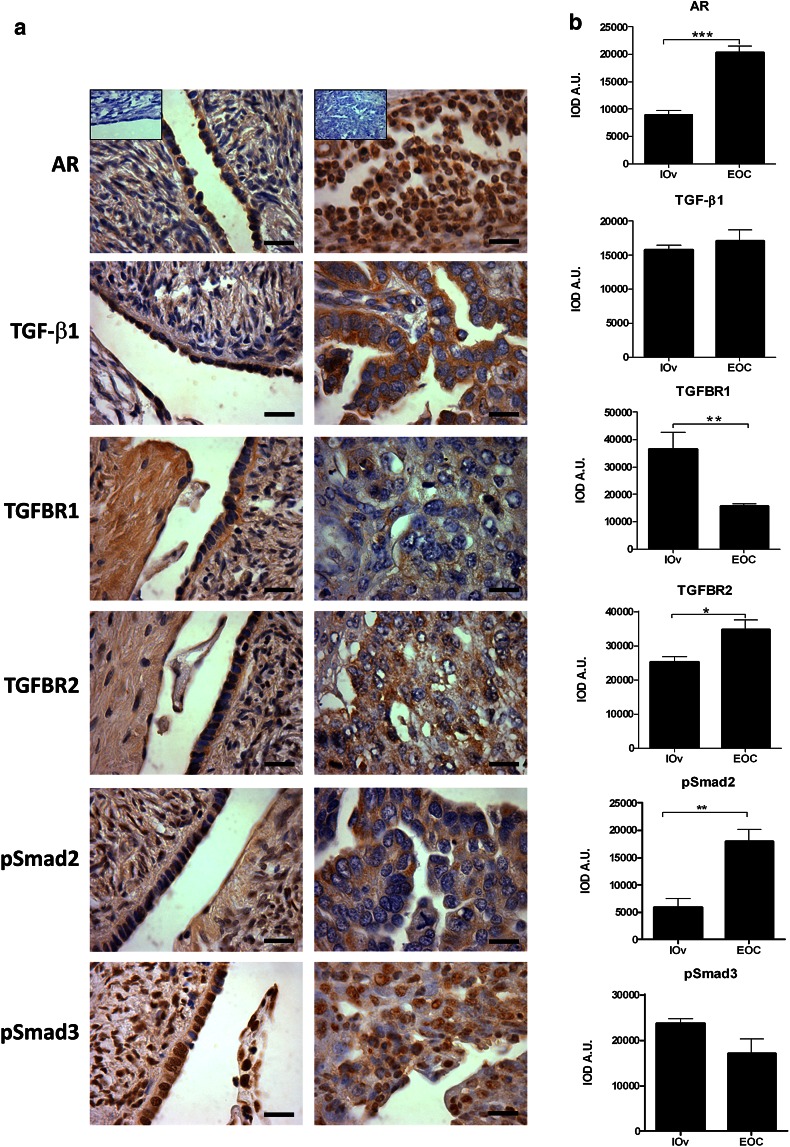
Immunodetection and semiquantitative analysis of AR, TGF-β1, its receptors (TGFBR1 and TGFBR2), pSmad2 and pSmad3 in ovarian tissue. In **a** detection for AR, TGF-β1, TGFBR1, TGFBR2, pSmad2 (Ser467), pSmad3 (Ser423 + Ser425) proteins in paraffin wax section of I-Ov tissue (*right panel*) and EOC tissue (*left panel*), *n* = 7 for each study group. Positive staining for each antigen (*brown color*) was detected in epithelial compartment of all studied ovarian samples. As a negative control, the primary antibody was omitted. **b** Represents the semiquantitative analysis. Immunostaining was expressed as mean ± SEM. *p < 0.05; **p < 0.01; ***p < 0.001. *Scale bar* represents 50 μm

The presence of AR was analyzed in I-Ov and poorly differentiated EOC specimens. Positive immunodetection was assessed for such protein in both tissue types (Fig. 1). A weak staining was observed for AR in the nuclei and the cytoplasm of epithelial cells of ovarian tissues I-Ov, while nuclear and cytoplasmic staining for AR was strong in EOC tissues (Fig. 1a). The semiquantitative analysis of AR protein levels showed a significant increase in this protein in EOC tissues as compared to I-Ov tissues (*p* < 0.001) (Fig. 1b).

Furthermore, the presence of TGF-β, its receptors (TGFBR1—TGFBR2) and Smad2/3 proteins in their phosphorylated status was analyzed in ovarian tissue. There were no differences in TGF-β1 immunodetection between both study groups. However, TGFBR1 showed a homogeneous cytoplasmic staining in I-Ov tissues, while in contraposition, TGFBR1 detection was weak in the cytoplasmic compartment of EOC samples (Fig. 1a). The semiquantitative analysis (IOD-AU) evidenced a significant decrease in positive staining for such receptor in EOC as compared to control tissues (*p* < 0.05) (Fig. 1b). On the other hand, when analyzing TGFBR2 levels, detection was positive in both studied ovarian tissue types, and the semiquantitative analysis evidenced a significant increase in TGFBR2 in EOC samples (*p* < 0.05) (Fig. 1b).

Other critical molecules implicated in TGF-β1 signaling pathway are the Smad proteins that are activated following phosphorylation. Immunohistochemical analysis was carried out for phosphorylated Smad2 and Smad3 with antibodies against their specific sites of phosphorylation by TGF-β1 receptor. Thus, Smad2 phosphorylation in Ser467 (pSmad2) and Smad3 phosphorylation in Ser423 + Ser425 (pSmad3) were analyzed. Figure 1a shows positive immunodetection for both proteins in I-Ov and EOC tissues. Both cytoplasmic and nuclear staining was observed for pSmad2 in I-Ov tissues, while staining was mainly cytoplasmic in EOC tissues. A significant increase in pSmad2 was found in EOC tissues as compared to I-Ov tissues (*p* < 0.05) (Fig. 1b). The pSmad3 protein was observed in both study tissues, showing a strong nuclear staining. The semiquantitative analysis did not show differences between I-Ov and EOC tissues (Fig. 1b). All together, the results herein show that the TGF-β1 signaling pathway is altered, suggesting changes in cell cycle control in EOC tissues.

Protein levels of AR, TGF-β1 and molecules of the TGF-β1 signaling pathway in ovarian epithelial cells.

**Fig. 2:**
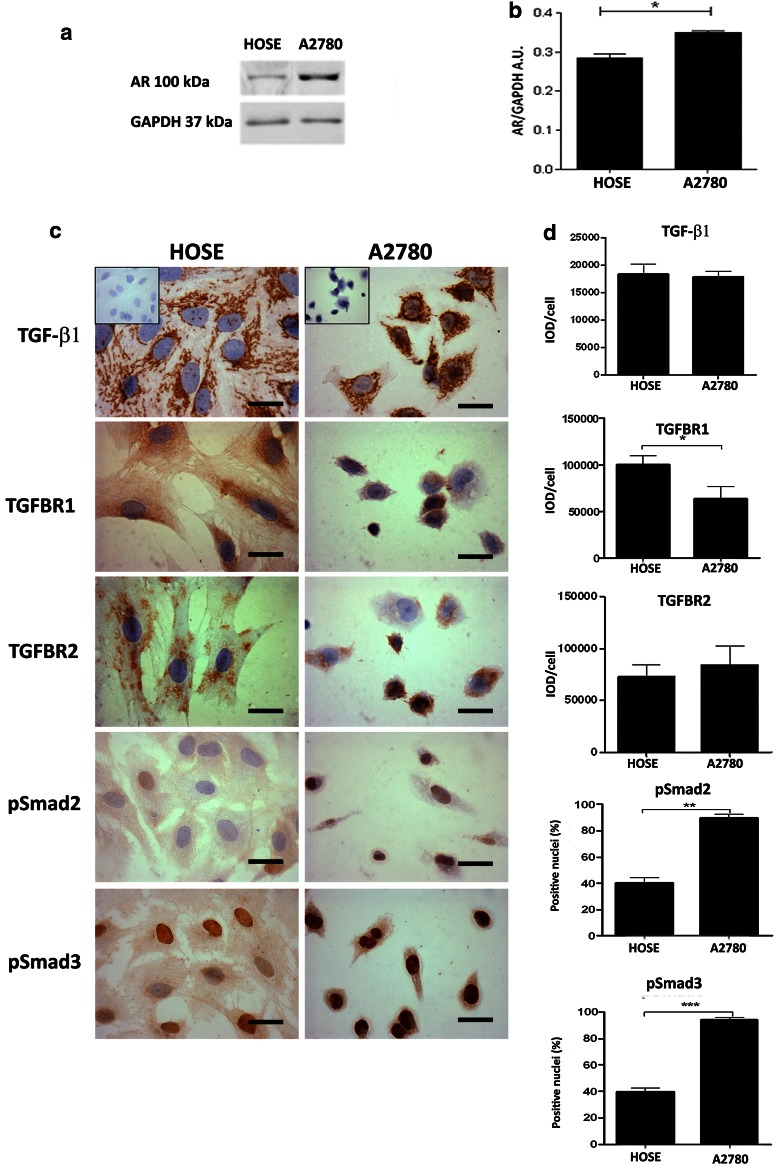
Immunodetection and semiquantitative analysis of AR, TGF-β1, its receptors (TGFBR1 and TGFBR2) and pSmad2 and pSmad3 in ovarian cell lines. **a** Western blotting analysis was performed for AR in cell lines, and equal amount of total protein for HOSE and A2780 cell lines was loaded in each lane. Protein AR was detected as bands with molecular mass of 110 kDa; **b** represents the semiquantitative analysis of three independent experiments for AR in arbitrary units (AU); **c** detection for TGF-β1, TGFBR1, TGFBR2, pSmad2 (Ser467), pSmad3 (Ser423 + Ser425) proteins in HOSE and A2780 cell lines. As a negative control, the primary antibody was omitted; **d** represents the semiquantitative analysis; 1000 cells were analyzed for each molecule in three independent experiments. Immunostaining was expressed as mean ± SEM. *p < 0.05; **p < 0.01; ***p < 0.001. *Scale bar* represents 20 μm

To address this objective, cellular location of TGF-β1 and its receptors was analyzed through immunocytochemistry (Fig. 2c, d). Androgen receptor levels were assessed through Western blot (Fig. 2a, b). A filamentous staining of TGF-β1 was observed in HOSE and A2780 cells. In HOSE cells, TGF-β1 immunostaining was homogeneous, while in A2780 cells, it was heterogeneous, and some cells were more strongly stained than others (Fig. 2c). Furthermore, AR protein levels were significantly lower in HOSE cells as compared to A2780 cells (*p* < 0.05) (Fig. 2a, b).

Cellular location of TGFBR1, TGFBR2 and Smad2/3 proteins was assessed in HOSE and A2780 cells (Fig. 2c). For TGFBR1, a strong granular cytoplasmic staining was detected in HOSE cells, while staining was weak in A2780 cells, thus evidencing a significant decrease in TGFBR1 levels in A2780 cells, as compared to HOSE cells (Fig. 2d). The presence of TGFBR2 was detected in both cell lines; however, there were no differences in TGFBR2 levels between HOSE and A2780 cells (Fig. 2d).

Smad proteins were also analyzed in the study cell lines (Fig. 2b). Positive immunodetection for pSmad2 was evidenced in both cell lines. Protein levels of pSmad2 were higher in A2780 cells. Conversely, staining for pSmad3 was mainly nuclear and significantly higher in A2780 cells, as compared to HOSE cells (*p* < 0.05) (Fig. 2c, d). Such results correlate adequately with the results obtained from ovarian tissue specimens in I-Ov and EOC, thus suggesting that HOSE and A2780 cell lines constitute a representative in vitro model to study epithelial ovarian cancer.

### Effect of DHT on mRNA levels and TGFBR1 and TGFBR2 protein levels on HOSE and A2780 cell lines

**Fig. 3:**
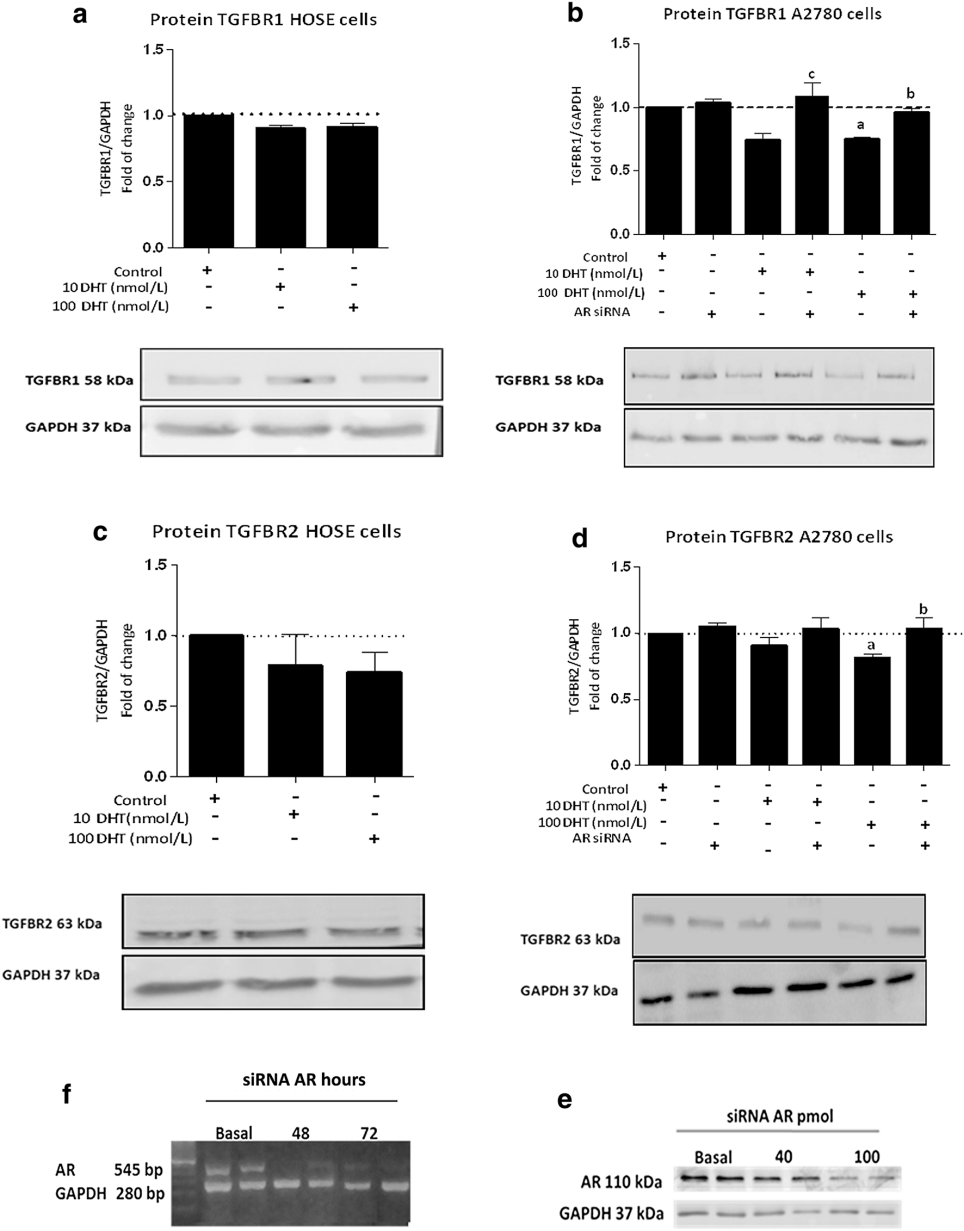
Effect of DHT on TGFBR1 and TGFBR2 protein levels in HOSE and A2780 cells. Cell cultures were exposed to 10 or 100 nmol/L DHT for 72 h; in addition, A2780 cells were treated with or without AR siRNA. As control, red phenol-free culture medium was used. Immunoblot and semiquantitative analysis of TGFBR1 in HOSE and A2780 cells (**a** and **b**, respectively) detected as a band with a molecular mass of 58 kDa. Immunoblot and semiquantitative analysis of TGFBR2 in HOSE and A2780 cells (**c** and **d**, respectively) detected as a band at 63 kDa. Conventional RT-PCR in different hours (**e**) and Western blotting analysis with different amounts of AR siRNA (**f**) were performed for AR. Band intensities were quantified by scanning densitometry and normalized to intensities observed for GAPDH (37 kDa) as internal control. The data from five independent experiments were expressed as mean of fold change respect to control ± SEM. **a** <0.05 100 nmol/L versus control; **b** <0.05 100 nmol/L versus AR siRNA; **c** <0.05 10 nmol/L versus AR siRNA

Because TGFBR1 and TGFBR2 activation is important in TGF-β1 signaling pathway, we assessed whether such receptor levels underwent changes as a result of different DHT doses and different exposure times. Both TGF-β1 receptors were analyzed in A2780 cells after treatment of 10 and 100 nmol/L DHT during 48 h. No differences were detected in TGFBR1 and TGFBR2 mRNA and protein levels (data not shown). Moreover, when A2780 were treated with 100 nmol/L DHT during 72 h, a significant decrease in TGFBR2 mRNA levels was observed, as compared to the control condition (control = 1.1 ± 0.0; 10 nmol/L DHT = 0.9 ± 0.017; 100 nmol/L DHT = 0.87 ± 0.01 *; * *p* < 0.05 vs. control); there were no differences between study groups in TGFBR1 mRNA levels (control = 1.0 ± 0.0; 10 nmol/L DHT = 0.95 ± 0.06; 100 nmol/L DHT = 0.87 ± 0.03; *p* = 0.11). However, a significant decrease in TGFBR1 (Fig. 3b) and TGFBR2 (Fig. 3d) protein levels was observed in A2780 cells treated with 100 nmol/L DHT (*p* < 0.05). Contrariwise, treatment with DHT did not have any effect on HOSE cells (Fig. 3a–c).

A siRNA against AR was used to assess whether the decrease in TGFBR1 and TGFBR2 protein levels in A2780 cells was an effect of the AR-DHT complex. The AR siRNA reduced AR protein levels in 45 % (Fig. 3f), in turn, non-specific siRNA (*n*-siRNA) did not modify AR levels (control = 1 ± 0.0; *n*-siRNA = 1.07 ± 0.01; *p* = 0.08). Whether AR silencing was maintained during the 72 h of stimulation was also assessed (Fig. 3e). Between 48 and 72 h, AR levels underwent knockdown, while at the fifth day, AR mRNA levels were recovered (Fig. 3e). Then, A2780 transfected cells with and without AR siRNA were treated with 10 and 100 nmol/L DHT to analyze TGFBR1 and TGFBR2 protein levels. A decrease in TGFBR1 and TGFBR2 protein levels was evidenced with DHT; however, in the presence of the AR siRNA, receptor protein levels were similar to those of control conditions with 100 nmol/L DHT (Fig. 3b–d). These results indicate that the DHT-AR complex might be acting at the transcriptional or translational level and might cause, by this means, a decrease in TGFBR1 and TGFBR2 protein levels.

### Effect of TGF-β1 and DHT on p21 protein levels in ovarian epithelial cells

**Fig. 4:**
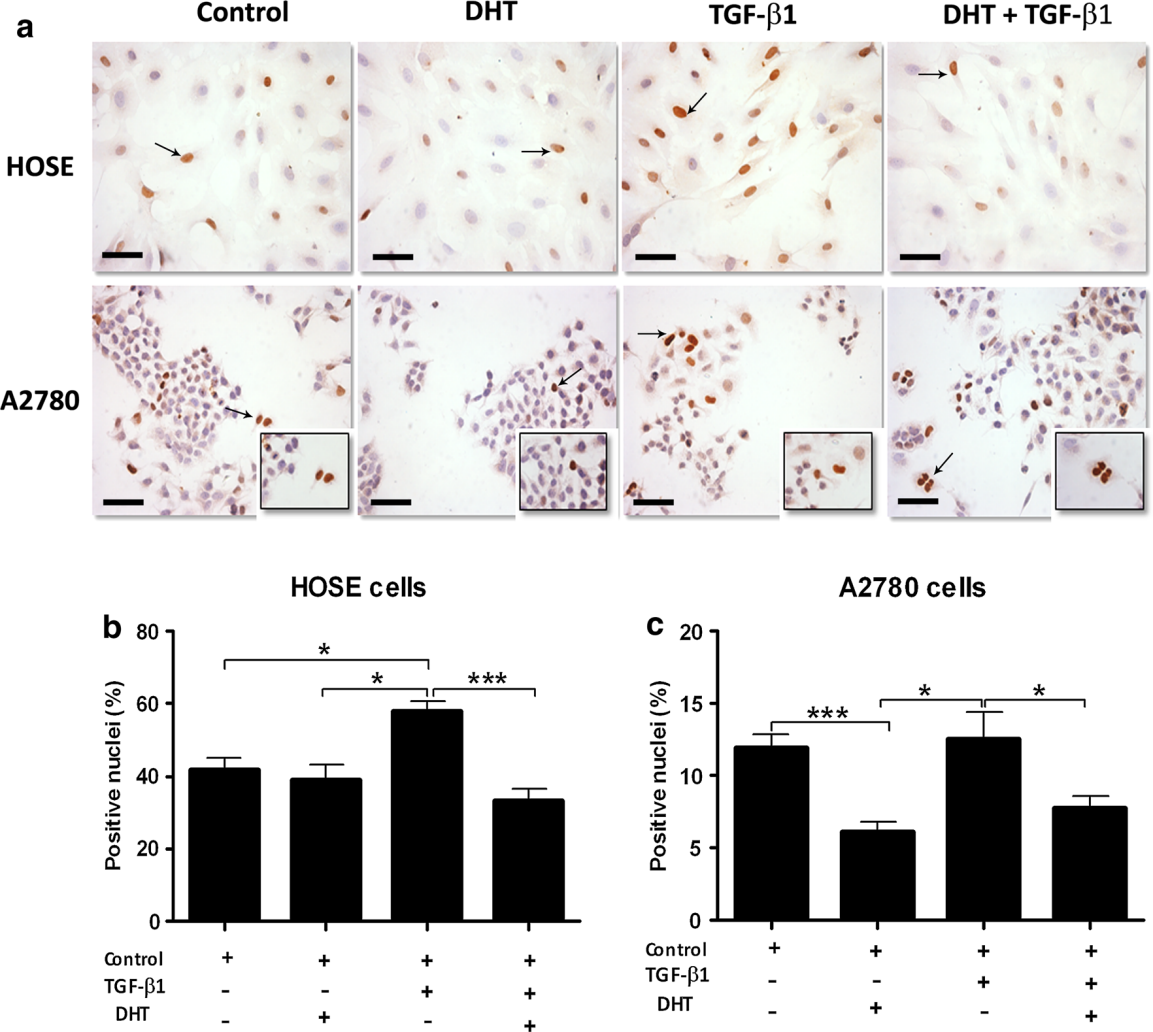
Immunodetection and semiquantitative analysis of p21 protein levels in ovarian cell lines. HOSE and A2780 cell lines were treated for 72 h with 100 nmol/L DHT, 10 ng/mL TGF-β1 and DHT plus TGF-β1 and controls with only culture medium. **a** Shows a representative microphotography, where positive staining (*brown color*) was detected in the nuclei. The semiquantitative analysis is presented in **b** for HOSE cell line and **c** for A2780 cell line. Immunostaining was measured by the percentage of positive nuclei. The results were expressed as positive nuclei % ± SEM. *p < 0.05; ***p < 0.001. *Scale bar* represents 50 μm

To assess whether the decrease in TGFBR1 and TGFBR2 levels caused by DHT had an impact on the response of the TGF-β1 signaling pathway, levels of the cell cycle inhibitor p21 were studied in HOSE and A2780 cells after administration of 10 ng/mL TGF-β1 and 100 nmol/L DHT during 72 h (Fig. 4). Levels of p21 were analyzed with immunocytochemistry, and the percentage of positive nuclei was obtained in HOSE and A2780 cells (Fig. 4a). There were no changes in the percentage of p21-positive nuclei in HOSE cells in presence of DHT as compared to control conditions (Fig. 4a, b). However, in the presence of TGF-β1, there was a significant increase in the percentage of p21-positive nuclei in HOSE cells (*p* < 0.05). Moreover, when such cells were treated with DHT plus TGF-β1, there were no differences as compared to controls (Fig. 4a, b). As for A2780 cells, in control conditions, there was a lower percentage of p21-positive nuclei as compared to HOSE cells in the same conditions (Fig. 4a). In the presence of DHT, the percentage of p21-positive nuclei among A2780 cells decreased significantly (*p* < 0.001) as compared to A2780 cell controls (Fig. 4a–c). On the other hand, in the presence of TGF-β1, the percentage of p21-positive nuclei did not increase as expected; p21 levels were similar to control conditions. Interestingly, there was a significant decrease in p21 as compared to control conditions (*p* < 0.05) when A2780 cells were treated with DHT plus TGF-β1 (Fig. 4a–c). These results suggest that DHT might be disrupting the TGF-β1 signaling pathway, thus affecting p21 expression. Therefore, the latter might result in a modification of the cell cycle in epithelial ovarian cancer cells.

## Discussion

In several pathological conditions, including cancer, there are defects in several growth factor signaling pathways, including TGF-β (Siegel and Massagué 2003; Vera et al. 2014). The possible mechanisms that might be inhibiting the anti-proliferative action of TGF-β still remain unclear. In order to address this issue, the present research examined protein levels of the molecules implicated in the TGF-β signaling pathway such as TGF-β, its receptors and Smad2/3 proteins in tissues from patients with EOC and also in ovarian cell lines. Additionally, the possible role of androgens in TGF-β signaling pathway was analyzed. Our group and others (Elattar et al. 2012; Gibson et al. 2014) have demonstrated an increase in AR protein in EOC that in turn indicates a potential susceptibility of such tissue to androgen action, as it has been found in other tissues, such as the prostate (Zhou et al. 2015). Chipuk et al. (2002) described the interaction between androgens and the TGF-β signaling pathway. Through this interaction, androgens are able to block TGF-β response in epithelial cells of the prostate. Such inhibition might be exerted through an association between AR and Smad3, thus preventing the binding of Smad3 to Smad-binding elements (SBE) y, consequently, blocking the transcription of genes related to cell growth inhibition. According to our results, TGF-β1 protein levels were not modified either in EOC or in the studied cell lines, thus suggesting that the failure in cell cycle control might be at the downstream molecules of the TGF-β1 cascade, such as its receptors or Smad proteins. In this regard, the present work found a decrease in TGFBR1 protein levels in ovarian cancer tissue, thus suggesting that DHT might downregulate TGFBR1 protein levels but not the mRNA levels of this receptor. There is a little information about the androgens action in TGFBR1 levels regulation in other tissues. Nevertheless, some studies have shown that TGFBR1 expression may be regulated by the micro-RNA-141, among other micro-RNAs (Butz et al. 2012; Denby et al. 2011). Additionally, other studies have shown that androgens are able to regulate miR-141 levels (Tiryakioglu et al. 2013; Waltering et al. 2011). Consequently, it is tempting to propose that the decrease in TGFBR1 that was found in the present study might represent a posttranscriptional effect of androgens, although more information is needed to establish such effect. On the other hand, we found an increase in TGFBR2 protein levels in ovarian cancer tissue, but not in the A2780 cell line. However, it is important to remember that the signaling pathway begins when TGF-β binds firstly to TGFBR2 that activates and recruits TGFBR1. This latter molecule is responsible for phosphorylation and activation of Smad proteins (Siegel and Massagué 2003). According to the results obtained in the present study, TGFBR1 was decreased in EOC tissues, suggesting that Smad protein activation and thus the response of the signaling pathway are impaired, and therefore, the increase in TGFBR2 that was found might be a compensatory effect of the tissue to balance the lack of response of such signaling pathway.

Discrepancies that were evidenced between the cell line and the tissue might also reside in the activation of other signaling pathways present in the tissue, in which TGFBR2 might participate, such as MAPK activation and other Smad-independent signaling pathways implicated in the proliferation process (Derynck and Zhang 2003). Another important point to take into consideration is that TGBR2 expression is also regulated by different micro-RNAs (Butz et al. 2012) that have not been fully analyzed in the ovary. Therefore, a study of these micro-RNAs in ovarian cancer might contribute to the understanding of the differences observed between the cell line and the tissue.

Also, treatment with DHT in A2780 cancer cell line resulted in a decrease in mRNA levels and in TGFBR2 protein levels, consistent with studies in other ovarian cell lines (Evangelou et al. 2000). The mechanism through which DHT acts is poorly understood. Some evidences in prostate cancer have demonstrated that androgens may downregulate TGFBR2 gene expression through a transcriptional mechanism in which DHT suppresses the binding of the transcription factor Sp1 to the TGFBR2 promoter. Levels of Smad3 mRNA could be reduced by the same mechanism (Song et al. 2008, 2010). In addition, other studies in prostate cancer demonstrated that AR may regulate the decrease in TGFBR2 levels through micro-RNA-21 (Mishra et al. 2014).

The other molecules of the TGF-β signaling pathway such as Smad proteins are also deregulated in EOC. An increase in Smad2 phosphorylation was detected in EOC; however, its cellular location was mainly cytoplasmic rather than nuclear. It has been stated that Smad protein build-up in the nucleus is closely related to TGF-β receptor activity (Inman et al. 2002). Moreover, mutations in Smad2 have been described to cause a loss of affinity of this protein for certain nucleoporins, with a concomitant failure in translocation of Smad2 toward the nucleus (Xu et al. 2002). On the other hand, increased phosphorylated Smad3 at a nuclear location in ovarian cancer cells observed in the present study, does not necessarily indicate that Smad3 could be involved in cell cycle inhibition. With regard to the latter, there are other phosphorylation sites for Smad3, the linker region, which may allow the activation of processes such as cell growth and invasion (Matsuzaki 2011). In fact, a study suggests that phosphorylation at the linker region of Smad3 does not affect either its activation by TGF-β or its translocation to the nucleus, but would affect the transcriptional activity of Smad3 and would block the expression of the inhibitor of the cell cycle such as p15 (Choi et al. 2013). Phosphorylation in this region could be induced by reactive oxygen species (ROS) such as hydrogen peroxide (H2O2) that activates the Akt-ERK1/2-linker signaling pathway (Choi et al. 2013). In ovarian cancer, it has been demonstrated that growth factors such as EGF may induce the production of H_2_O_2_ (Cheng et al. 2010), while in other cell types, it has also been observed that NGF can cause an increase in H_2_O_2_ production (Chiba et al. 2014). It is important to highlight that EGF and NGF are overexpressed in ovarian cancer (Bartlett et al. 1996; Campos et al. 2007; Tapia et al. 2011). Therefore, it is possible to propound that in ovarian cancer, H_2_O_2_ might induce Smad3 phosphorylation at the linker region. Nevertheless, the mechanism should be confirmed in other studies.

Finally, to assess the response of the e TGF-β signaling pathway in ovarian cells, protein levels of the cell cycle inhibitor p21 were analyzed. Data of the present research reveal that under treatment with DHT, p21 protein levels decrease in ovarian cancer, suggesting a failure of TGF-β1 response in ovarian cancer cells. As it is known, p21 is a cyclin-dependent kinase protein (CDK), an inhibitor belonging to the Cip/Kip family of CDK inhibitors. Activation of p21 is crucial to cell cycle progression (Gartel and Radhakrishnan 2005). Several transcription factors such as Sp1/Sp3 (Gartel et al. 2001) regulate p21 levels. Sp1/Sp3 transcription factors are regulated by DHT (Song et al. 2008), thus indicating that a decrease in p21 protein levels may be a result of a direct effect of androgens or a consequence of the failure of the TGF-β response due to an androgen-induced decrease in TGF-β receptors.

In conclusion, the present research evaluated the expression of molecules involved in the TGF-β-Smads signaling pathway and their association to androgens. The results obtained in EOC tissue and in the A2780 cell line, together with the studies of other authors carried out in other ovarian cell lines, suggest that the canonical TGF-β signaling pathway might be altered in EOC where androgens may play an important role in downregulating receptor expression, particularly TGFBR1. The latter might be potentially involved in decreasing p21 levels. Additionally, androgens may act directly on p21 expression by inducing their decrease. Such defects, among others, might contribute to epithelial proliferation in ovarian cancer, and their further study is necessary to elucidate the implicated mechanisms.
